# Comparison of 4 Acute Pulmonary Embolism Mortality Risk Scores in Patients Evaluated by Pulmonary Embolism Response Teams

**DOI:** 10.1001/jamanetworkopen.2020.10779

**Published:** 2020-08-26

**Authors:** Geoffrey D. Barnes, Alona Muzikansky, Scott Cameron, Jay Giri, Gustavo A. Heresi, Wissam Jaber, Todd Wood, Thomas M. Todoran, D. Mark Courtney, Victor Tapson, Christopher Kabrhel

**Affiliations:** 1 Frankel Cardiovascular Center, Department of Internal Medicine, University of Michigan, Ann Arbor; 2 Biostatistics Center, Massachusetts General Hospital, Boston; 3 Department of Cardiovascular Medicine, Cleveland Clinic, Cleveland, Ohio; 4 Department of Internal Medicine, University of Pennsylvania, Philadelphia; 5 Department of Pulmonary and Critical Care Medicine, Cleveland Clinic, Cleveland, Ohio; 6 Division of Cardiology, Department of Internal Medicine, Emory University, Atlanta, Georgia; 7 Division of Cardiology, Department of Internal Medicine, Lancaster General Hospital, Lancaster, Pennsylvania; 8 Division of Cardiovascular Medicine, Department of Internal Medicine, Medical University of South Carolina, Charleston; 9 Department of Emergency Medicine, University of Texas Southwestern, Dallas; 10 Division of Pulmonary and Critical Care Medicine, Department of Internal Medicine, Cedars-Sinai Hospital, Los Angeles, California; 11 Department of Emergency Medicine, Massachusetts General Hospital, Boston

## Abstract

**Importance:**

The risk of death from acute pulmonary embolism can range as high as 15%, depending on patient factors at initial presentation. Acute treatment decisions are largely based on an estimate of this mortality risk.

**Objective:**

To assess the performance of risk assessment scores in a modern, US cohort of patients with acute pulmonary embolism.

**Design, Setting, and Participants:**

This multicenter cohort study was conducted between October 2016 and October 2017 at 8 hospitals participating in the Pulmonary Embolism Response Team (PERT) Consortium registry. Included patients were adults who presented with acute pulmonary embolism and had sufficient information in the medical record to calculate risk scores. Data analysis was performed from March to May 2020.

**Main Outcomes and Measures:**

All-cause mortality (7- and 30-day) and associated discrimination were assessed by the area under the receiver operator curve (AUC).

**Results:**

Among 416 patients with acute pulmonary embolism (mean [SD] age, 61.3 [17.6] years; 207 men [49.8%]), 7-day mortality in the low-risk groups ranged from 1.3% (1 patient) to 3.1% (4 patients), whereas 30-day mortality ranged from 2.6% (1 patient) to 10.2% (13 patients). Among patients in the highest-risk groups, the 7-day mortality ranged from 7.0% (18 patients) to 16.3% (7 patients), whereas 30-day mortality ranged from 14.4% (37 patients) to 26.3% (26 patients). Each of the risk stratification tools had modest discrimination for 7-day mortality (AUC range, 0.616-0.666) with slightly lower discrimination for 30-day mortality (AUC range, 0.550-0.694).

**Conclusions and Relevance:**

These findings suggest that commonly used risk tools for acute pulmonary embolism have modest estimating ability. Future studies to develop and validate better risk assessment tools are needed.

## Key Points

**Question:**

How well do different risk assessment tools estimate 7- and 30-day mortality in patients with acute pulmonary embolism?

**Findings:**

This cohort study of 416 patients with acute pulmonary embolism found that commonly used risk assessment tools have only moderate discriminative ability for 7- and 30-day mortality in patients with acute pulmonary embolism.

**Meaning:**

These findings suggest that clinicians may need to integrate broad clinical information rather than relying on a single risk assessment tool to estimate mortality risk and determine management for patients with acute pulmonary embolism.

## Teaser

This cohort study examines the performance of risk assessment scores among US patients with acute pulmonary embolism.

## Introduction

Most patients with acute pulmonary embolism (PE) experience few, if any, complications. However, an estimated 5% to 15% of patients with acute PE are at high risk of death or hemodynamic collapse.^1^ Multiple risk assessment models have been developed to identify patients at risk for these complications. These commonly used risk scores are widely recommended in various treatment guidelines and expert recommendations.^2,3^

Some risk scores are primarily used to identify low-risk patients for whom outpatient treatment may be appropriate.^4,5^ Others aim to identify patients for whom the risk of death and hemodynamic deterioration is sufficiently high to consider the use of thrombolytic or other advanced therapies. Common examples include the Pulmonary Embolism Severity Index (PESI), the simplified PESI (sPESI), and Bova scores.^6,7^ The PESI and sPESI have demonstrated the ability to discriminate between high and low risk for 30-day all-cause mortality.^2,8,9^ However, they were derived from retrospective cohorts and may not be ideal for guiding clinical decision-making at the time of PE diagnosis. A fourth system, outlined in the 2014 and 2019 European Society of Cardiology (ESC) guidelines,^2,10^ recommends using PESI or the sPESI but also adds biomarker and radiological markers to the risk stratification scheme.

It is unknown how similarly each of these risk tools will stratify an individual patient’s risk. In addition, inconsistent ability to estimate shorter-term outcomes (eg, 7- and 30-day mortality) limits the utility of many of these risk scores.^11^ Our objective was to explore the short-term outcomes among patients with acute PE and to compare the ability of different risk scores to discriminate among low- and high-risk patients.

## Methods

At 8 participating Pulmonary Embolism Response Team (PERT) Consortium Pilot Registry hospitals, patients with acute PE were referred for a PERT evaluation by clinical specialists. Institutional review board approval for a waiver of informed consent was obtained at each center because the study was deemed to pose minimal risk to patients.

Criteria for PERT consultation vary at each center but generally require a radiographically confirmed acute PE in an adult patient for whom the primary team has some question about PE-related management. PERT consultation typically involved discussions between the primary team and 1 or more PE experts to help assess PE-related risk and guide management decision-making.^12,13^ Independent of clinical care, data were abstracted from the medical record into the PERT Consortium Pilot Registry using predefined data elements and a REDCap database. Included patients presented between October 2016 and October 2017. Abstraction at each center was performed by a physician or trained research staff member. Full details of the registry have been published elsewhere.^14^

### Risk Scores

We calculated the PESI, sPESI, and Bova scores for each patient with confirmed PE in the PERT Consortium pilot registry who had complete data required to calculate each risk score, using the methods from the derivation studies (eFigures 1, 2, 3, 4, and 5 in the Supplement).^6,7,8^ We also assigned an ESC risk to each patient according to the presence of shock or hypotension (defined as systolic blood pressure [SBP] <90 mm Hg), signs of right ventricular dysfunction on echocardiogram or computed tomography, and elevated cardiac biomarkers.^2^ We did not include the PESI or sPESI score in the ESC risk stratification to avoid collinearity when comparing ESC with PESI or sPESI.

In addition to calculating individual PESI, sPESI, and Bova scores, we categorized patients according to the risk categories from the derivation studies.^6,7,8^ For PESI, we included 5 risk classes (I-V), whereas for sPESI we included a low risk (score = 0) and high risk (score >0). The Bova score categorizes patients into 3 risk groups (I-III) but excludes patients presenting with hypotension. To properly compare an individual’s risk between different scores, we modified the Bova score using 2 methods. First, we created a fourth risk group for patients with an SBP less than 90 mm Hg at presentation. Second, we included all patients with SBP less than 90 mm Hg into the high-risk group (class III).

Patient survival was assessed at standard intervals in the PERT Consortium pilot registry. We assessed for all-cause death at any time from hospital presentation to 7 days and up to 30 days. We addressed missing data using multiple imputation. Twenty-five data sets were imputed using the fully conditional specification method with logistic regression for class variable and estimated mean matching for continuous variables. These data sets were then used for the construction of the predictive logistic regression models, and the results from the multiple models were pooled for analysis using the inferential multiple imputation analysis strategy by Rubin.^15^

### Statistical Analysis

The discrimination of each risk score to estimate 7- and 30-day mortality was calculated by measuring the area under the receiver operator curve (AUC) and was compared using a contrast matrix approach.^16^ A 2-sided *P* < .05 was considered significant. All statistical analyses were performed using SAS statistical software version 9.4 (SAS Institute). Data analysis was performed from March to May 2020.

## Results

Our analysis includes 416 adult patients (mean [SD] age, 61.3 [17.6] years; 207 men [49.8%]; mean [SD] body mass index [calculated as weight in kilograms divided by height in meters squared], 31.9 [10.1]) with acute PE presenting to 8 hospitals (Table 1). Nearly one-half of the patients received anticoagulation therapy alone (188 patients [45.2%]), with fewer receiving thrombolysis (32 patients [7.7%]), thrombectomy (11 patients [2.6%]), or other advanced interventions (106 patients [25.5%]).

**Table 1.** Patient Characteristics

Characteristic^a^	Patients, No. (%)		
	Survivors (n = 365)	Nonsurvivors (n = 51)	Total (N = 416)
Demographic characteristics			
Age, mean (SD), y	61.0 (17.7)	63.2 (16.8)	61.3 (17.6)
Male	182 (49.9)	25 (49.0)	207 (49.8)
Race/ethnicity			
Asian	6 (1.6)	0	6 (1.4)
Black or African American	94 (25.8)	12 (23.5)	106 (25.5)
Hispanic or Latino	14 (3.8)	2 (3.9)	16 (3.9)
White	218 (59.7)	30 (58.8)	248 (59.6)
Other or unknown	33 (9.0)	7 (13.7)	40 (9.6)
Height, mean (SD), cm	170.7 (24.1)	166.6 (27.2)	170.2 (24.5)
Weight, mean (SD), kg	94.0 (29.8)	91.0 (28.9)	93.6 (29.7)
Body mass index, mean (SD)^b^	32.0 (10.2)	31.4 (9.5)	31.9 (10.1)
Primary insurance			
None or self-pay	13 (3.6)	1 (2.0)	14 (3.4)
Private insurance	125 (34.3)	18 (35.3)	143 (34.4)
Medicare	136 (37.3)	25 (49.0)	161 (38.7)
Medicaid	39 (10.7)	2 (3.9)	41 (9.9)
Military	1 (0.3)	1 (2.0)	2 (0.5)
Other or unknown	51 (14.0)	4 (7.8)	55 (13.2)
Comorbidities			
Chronic obstructive pulmonary disease	37 (10.1)	5 (9.8)	41 (10.1)
Connective tissue disease	8 (2.2)	2 (3.9)	10 (2.4)
Congestive heart failure	19 (5.2)	4 (7.8)	23 (5.5)
Coronary artery disease	54 (14.8)	6 (11.8)	60 (14.4)
Depression or anxiety	67 (18.4)	7 (13.7)	74 (17.8)
Diabetes	89 (24.4)	11 (12.6)	100 (24.0)
Gastrointestinal bleeding	10 (2.7)	2 (3.9)	12 (2.9)
Hypertension	211 (57.8)	31 (60.8)	242 (58.2)
Malignant neoplasm	87 (23.8)	24 (47.1)	111 (26.7)
Metastatic disease	34 (9.3)	12 (23.5)	46 (11.1)
Kidney insufficiency	33 (9.0)	3 (5.9)	36 (8.7)
Stroke or neurovascular disease	26 (7.1)	2 (3.9)	28 (6.7)
Charlson Comorbidity Index score, mean (SD)	1.9 (2.3)	2.8 (2.9)	2.0 (2.4)
Other pulmonary embolism risk factors			
Tobacco use	108 (29.6)	20 (39.2)	128 (30.8)
Recent hospitalization (within 4 wk)	81 (22.2)	16 (31.4)	97 (23.3)
Reduced mobility	55 (15.1)	10 (19.6)	65 (15.6)
Recent surgery or invasive procedure (within 4 wk)	71 (19.5)	13 (25.5)	84 (20.2)
Prior pulmonary embolism	59 (16.2)	8 (15.7)	67 (16.1)
Prior deep vein thrombosis	64 (17.5)	8 (15.7)	72 (17.3)
Family history of venous thromboembolism	29 (8.0)	1 (2.0)	30 (7.2)
Hormone use	27 (7.4)	1 (2.0)	28 (6.7)
Recent trauma (within 4 wk)	17 (4.7)	2 (3.9)	19 (4.6)
Indwelling vascular catheter	10 (2.7)	7 (13.7)	17 (4.1)
In-hospital treatment			
Anticoagulation only	175 (66.0)	13 (25.5)	188 (45.2)
Systemic thrombolysis	25 (9.4)	7 (13.7)	32 (7.7)
Catheter-based intervention	45 (17.0)	8 (15.7)	53 (12.7)
Surgical embolectomy	6 (2.3)	5 (9.8)	11 (2.6)
Extracorporeal membrane oxygenation	3 (1.1)	6 (11.8)	9 (2.2)
Inferior vena cava filter placement	38 (14.3)	6 (11.8)	44 (10.6)

^a^ Patient characteristics are shown by survivorship status at 30 days.

^b^ Body mass index is calculated as weight in kilograms divided by height in meters squared.

Among the 416 patients, all-cause death occurred within 7 days for 25 patients (6.0%) and within 30 days for 51 patients (12.3%). As seen in Table 2, 7-day mortality in the low-risk groups ranged from 1.3% (1 patient; sPESI) to 3.1% (4 patients; Bova), whereas 30-day mortality ranged from 2.6% (1 patient; PESI) to 10.2% (13 patients; Bova); the rate was 3.8% (3 patients) for sPESI. Among patients in the highest-risk groups, the 7-day mortality ranged from 7.0% (18 patients; sPESI) to 16.3% (7 patients; SBP <90 mm Hg in Bova), whereas 30-day mortality ranged from 14.4% (37 patients; sPESI) to 26.3% (26 patients; PESI). Distribution of patients according to risk scores is shown in eFigures 1, 2, 3, 4, and 5 in the Supplement.

**Table 2.** Pulmonary Embolism Risk Scores and Associated 7- and 30-Day Mortality

Risk score	All patients, No.	Death within 7 d			Death within 30 d		
		Patients, No. (%)	*P* value	AUC (95% CI)	Patients, No. (%)	*P* value	AUC (95% CI)
European Society of Cardiology risk score							
Low	64	1 (1.6)	.02	0.638 (0.598-0.679)	5 (7.8)	.02	0.592 (0.563-0.621)
Intermediate-low	109	5 (4.6)			13 (11.9)		
Intermediate-high	146	7 (4.8)			15 (10.3)		
High	51	8 (15.7)			12 (23.5)		
Unable to assess^a^	46	4 (8.7)			6 (13.0)		
Pulmonary Embolism Severity Index							
Class I	39	1 (2.6)	.02	0.652 (0.629-0.675)	1 (2.6)	<.001	0.694 (0.677-0.710)
Class II	55	1 (1.8)			4 (7.3)		
Class III	65	3 (4.6)			5 (7.7)		
Class IV	56	0			2 (3.6)		
Class V	99	12 (12.1)			26 (26.3)		
Unable to assess^a^	102	8 (7.8)			13 (12.7)		
Simplified Pulmonary Embolism Severity Index							
Low risk	78	1 (1.3)	.004	0.666 (0.640-0.693)	3 (3.8)	<.001	0.657 (0.644-0.671)
Not low risk	257	18 (7.0)			37 (14.4)		
Unable to assess^a^	81	6 (7.4)			11 (13.6)		
Bova							
Class I	128	4 (3.1)	.01	0.639 (0.603-0.675)	13 (10.2)	.06	0.567 (0.540-0.594)
Class II	99	5 (5.1)			9 (9.1)		
Class III	78	4 (5.1)			9 (11.5)		
Systolic blood pressure <90 mm Hg	43	7 (16.3)			11 (25.6)		
Class III and systolic blood pressure <90 mm Hg^b^	121	11 (9.1)	.05	0.616 (0.581-0.651)	20 (16.5)	.24	0.550 (0.526-0.575)
Unable to assess^a^	68	5 (5.1)			9 (13.2)		

Abbreviation: AUC, area under the receiver operator curve.

^a^ Patients without complete data in the original data set to calculate individual risk scores were categorized as unable to assess. *P* value and AUC were calculated using the multiple imputed data set.

^b^ *P* value and AUC were calculated for Bova with systolic blood pressure less than 90 mm Hg as both a separate class and incorporated into class III.

Each of the risk stratification tools had modest discrimination for 7-day mortality (AUC range, 0.616-0.666) with slightly lower discrimination for 30-day mortality (AUC range, 0.550-0.694). Mean differences in AUC between the different scores were small for 7-day mortality (≤0.05) but somewhat larger for 30-day mortality, especially when comparing the Bova score with either the PESI (AUC difference, 0.13-0.14) or sPESI (AUC difference, 0.09-0.11) scores (eTable in the Supplement).

## Discussion

Our analysis demonstrates modest discrimination and ability to estimate 7- or 30-day mortality for each of the PE-specific risk scores, most of which are better for estimation of the shorter term outcomes. Additionally, there is little association among the 4 acute PE–specific risk scores.

Although not universally used, risk scores are increasingly recommended by guidelines for initial assessment of patients presenting with acute PE.^2,3,17^ On the basis of these initial assessments, treatment options (eg, anticoagulation alone, thrombolysis, or thrombectomy) are considered. However, our data suggest that no single risk score is highly accurate or superior to another for estimating short-term (7-day) or slightly longer-term (30-day) mortality.

PE risk scores are often used in a variety of clinical situations. For instance, the sPESI score has been shown to identify a cohort of patients at very low risk for complications and health care resource utilization for whom outpatient treatment may be preferred.^18^ However, it has also been found to misclassify many patients into the high-risk category for which they may be hospitalized unnecessarily.^19^

A systematic review^20^ from 2016 identified 17 different risk assessment models for PE-associated mortality. Overall 30-day mortality rates in that meta-analysis were similar to those for our study population among low-risk patients according to both the PESI (2.3% vs 2.6%) and the sPESI (1.5% vs 3.8%) scores. However, 30-day mortality rates differed between the meta-analysis and our study among those in the highest-risk groups for the PESI score (11.4% vs 26.3%) but less so for the sPESI score (10.7% vs 14.4%). This may be associated, in part, with the inclusion in our population of only patients for whom a PERT evaluation was requested. It is possible that patients with multiple comorbidities but hemodynamically stable PE did not have a PERT evaluation and, therefore, were not included in our registry.

From a research and quality improvement standpoint, use of any single PE risk stratification tool may not be adequate to appropriately risk-adjust patient outcomes. This is particularly relevant when comparing clinical outcomes across hospitals or organizations. In addition, the limited ability of any single risk score to estimate mortality may limit its use in identifying patients most likely to benefit from advanced therapies. It is possible that currently available therapies (eg, catheter-directed thrombolysis) may offer mortality benefit only if the patients most at risk for complications can easily be identified. To date, this risk assessment has limited success demonstrating patient-oriented clinical benefit in both the short and long term.^21,22^

### Strengths and Limitations

Our analysis has a number of important strengths, including the use of multicenter data from a prospective registry with predefined data elements. However, not all cases of acute PE at each center were captured. This is particularly true for lower-risk PE cases, for which PERT is not typically activated. It is possible that some of these scores are better estimators of outcomes and have an association with low-risk patients with acute PE. Second, only adverse events that were recorded in the medical record from the index hospital were recorded. In addition, mortality was assessed as all-cause rather than PE-specific because we are unable to assess death attributable to PE or to underlying comorbidities in the data set. Third, clinicians may have incorporated 1 or more of these scores into their management decisions, which potentially is associated with mortality outcomes. Fourth, larger validations studies are needed given the small overall number of deaths in our study population. However, we believe this to be one of the largest validation studies of these risk tools published to date. Fifth, we were unable to assess clinical gestalt as a factor associated with death, despite prior studies demonstrating good clinical estimation.^23^ Similarly, we are unable to assess for patient preference for different treatments because of limitations in the available data. Sixth, because of variation in the number of patients contributed from each center, including at least 1 center without any recorded deaths, we were unable to adjust for clustering at the health center level in our statistical models. Furthermore, central adjudication of data collection and adverse events was not able to be performed.

## Conclusions

These limitations notwithstanding, our analysis demonstrates only modest discrimination and ability to estimate short-term mortality in patients with acute PE. Furthermore, there appears to be minimal association among the different risk scores for individual patients. Future studies to develop and validate better risk assessment tools would improve both clinical care and PE-related research.

## Notes

Supplement. **eFigure 1.** European Society of Cardiology Risk Categories vs PESI Class **eFigure 2.** European Society of Cardiology Risk Categories vs Simplified PESI Class **eFigure 3.** European Society of Cardiology Risk Categories vs Bova Class **eFigure 4.** PESI Class vs Simplified PESI Class **eFigure 5.** Simplified PESI Class vs Bova Class **eTable. **Differences in Predictive Model Discrimination
