# Supplementary material for: Comparison of 4 Acute Pulmonary Embolism Mortality Risk Scores in Patients Evaluated by Pulmonary Embolism Response Teams
Source: JAMA Netw Open. 2020 Aug 26;3(8):e2010779. doi: 10.1001/jamanetworkopen.2020.10779 (PMC7450352; doi:10.1001/jamanetworkopen.2020.10779)
Supplement: Supplement. — eFigure 1. European Society of Cardiology Risk Categories vs PESI Class eFigure 2. European Society of Cardiology Risk Categories vs Simplified PESI Class eFigure 3. European Society of Cardiology Risk Categories vs Bova Class eFigure 4. PESI Class vs Simplified PESI Class eFigure 5. Simplified PESI Class vs Bova Class eTable. Differences in Predictive Model Discrimination [file jamanetwopen-3-e2010779-s001.pdf]

Supplementary Online Content

Barnes GD, Muzikansky A, Cameron S, et al. Comparison of 4 acute pulmonary embolism mortality risk scores in patients evaluated by pulmonary embolism response teams. *JAMA Netw Open*. 2020;3(8):e2010779. doi:10.1001/jamanetworkopen.2020.10779

eFigure 1. European Society of Cardiology Risk Categories vs PESI Class
eFigure 2. European Society of Cardiology Risk Categories vs Simplified PESI Class
eFigure 3. European Society of Cardiology Risk Categories vs Bova Class
eFigure 4. PESI Class vs Simplified PESI Class
eFigure 5. Simplified PESI Class vs Bova Class
eTable. Differences in Predictive Model Discrimination

This supplementary material has been provided by the authors to give readers additional information about their work.

© 2020 Barnes GD et al. *JAMA Network Open.*

To compare the distribution of risk stratification between scoring systems, we present the following heat maps to help visualize how individual patients are risk stratified. Of note, only patients with complete data to compute both comparative risk scores are included in each heat map.

eFigure 1. European Society of Cardiology Risk Categories vs PESI Class

ESC Risk Category	PESI Class: I (=65)	II (66-85)	III (86-105)	IV (106-125)	V (125+)
Low	10	13	11	6	10
Intermediate-Low	16	16	18	13	26
Intermediate-High	9	18	33	25	20
High	1	2	0	6	32

eFigure 2. European Society of Cardiology Risk Categories vs Simplified PESI Class

ESC Risk Category	sPESI Categories: Low (0)	Not Low Risk (≥1)
Low	19	33
Intermediate-Low	26	67
Intermediate-High	26	85
High	0	46

eFigure 3. European Society of Cardiology Risk Categories vs Bova Class

ESC Risk Category	BOVA Class: 1	2	3	SBP < 90		1	2	3 & SBP < 90
Low	52	3	0	0		52	3	0
Intermediate-Low	64	34	6	0		64	34	6
Intermediate-High	11	61	69	0		11	61	69
High	1	1	3	43		1	1	46

eFigure 4. PESI Class vs Simplified PESI Class

sPESI Category	PESI Class: I (≤65)	II (66-85)	III (86-105)	IV (106-125)	V (≥125)
Low (0)	26	33	15	0	2
Not Low (≥1)	13	22	50	56	97

© 2020 Barnes GD et al. *JAMA Network Open.*

eFigure 5. Simplified PESI Class vs Bova Class

		Bova Class							
		1	2	3	SBP < 90		1	2	3 & SBP < 90
sPESI Category	Low Risk (0)	48	22	0	0		48	22	0
	Not Low Risk (≥1)	61	66	59	40		61	66	99

eTable. Differences in Predictive Model Discrimination

	7-day Mortality Prediction		30-day Mortality Prediction	
	**Mean AUC Difference**	**95% CI**	**Mean AUC Difference**	**95% CI**
ESC vs. PESI	0.01	-0.12, 0.15	0.10	0.00, 0.20
ESC vs. sPESI	0.03	-0.10, 0.16	0.07	-0.03, 0.16
ESC vs. Bova (4 classes)	0.00	-0.08, 0.08	0.03	-0.08, 0.03
ESC vs. Bova (3 classes)	0.02	-0.11, 0.06	0.04	-0.10, 0.02
PESI vs. sPESI	0.01	-0.07, 0.09	0.04	-0.10, 0.03
PESI vs. Bova (4 classes)	0.01	-0.14, 0.11	0.13	0.02, 0.23
PESI vs. Bova (3 classes)	0.04	-0.16, 0.09	0.14	0.04, 0.25
sPESI vs. Bova (4 classes)	0.03	-0.08, 0.13	0.09	0.01, 0.17
sPESI vs. Bova (3 classes)	0.05	-0.05, 0.15	0.11	0.02, 0.19

Mean difference and 95% CI are presented for comparison between difference risk prediction scores for outcomes of 7- and 30-day mortality. Bova (4 classes) includes patients with systolic blood pressure <90mmHg as a separate class while Bova (3 classes) lumps patients with systolic blood pressure <90mmHg in with Class 3. AUC – area under the receiver-operator curve; ESC – European Society of Cardiology; PESI – Pulmonary Embolism Severity Index; sPESI – simplified PESI.

© 2020 Barnes GD et al. *JAMA Network Open.*
